# A Multi-Channel Multi-Scale Spatiotemporal Convolutional Cross-Attention Fusion Network for Bearing Fault Diagnosis

**DOI:** 10.3390/s25185923

**Published:** 2025-09-22

**Authors:** Ruixue Li, Guohai Zhang, Yi Niu, Kai Rong, Wei Liu, Haoxuan Hong

**Affiliations:** 1 College of Agricultural Engineering and Food Science, Shandong University of Technology, Zibo 255200, China; 23503060431@stumail.sdut.edu.cn (R.L.); 23503060443@stumail.sdut.edu.cn (Y.N.); 23503060439@stumail.sdut.edu.cn (K.R.); 23503060435@stumail.sdut.edu.cn (W.L.); 23503060441@stumail.sdut.edu.cn (H.H.); 2 Institute of Modern Agricultural Equipment, Shandong University of Technology, Zibo 255200, China

**Keywords:** multi-sensor information fusion, acoustic-vibration signals, multi-scale temporal convolution module, multi-feature extraction block, dual cross-attention module, fault diagnosis

## Abstract

Bearings, as commonly used elements in mechanical apparatus, are essential in transmission systems. Fault diagnosis is of significant importance for the normal and safe functioning of mechanical systems. Conventional fault diagnosis methods depend on one or more vibration sensors, and their diagnostic results are often unsatisfactory under strong noise interference. To tackle this problem, this research develops a bearing fault diagnosis technique utilizing a multi-channel, multi-scale spatiotemporal convolutional cross-attention fusion network. At first, continuous wavelet transform (CWT) is applied to convert the raw 1D acoustic and vibration signals of the dataset into 2D time–frequency images. These acoustic and vibration time–frequency images are then simultaneously fed into two parallel structures. After rough feature extraction using ResNet, deep feature extraction is performed using the Multi-Scale Temporal Convolutional Module (MTCM) and the Multi-Feature Extraction Block (MFE). Next, these traits are input into a dual cross-attention mechanism module (DCA), where fusion is achieved using attention interaction. The experimental findings validate the efficacy of the proposed method using tests and comparisons on two bearing datasets. The testing findings validate that the suggested method outperforms the existing advanced multi-sensor fusion diagnostic methods. Compared with other existing multi-sensor fusion diagnostic methods, the proposed method was proven to outperform the five existing methods (1DCNN-VAF, MFAN-VAF, 2MNET, MRSDF, and FAC-CNN).

## 1. Introduction

As industrial automation levels advance, bearings, as one of the important components in various mechanical devices, and their state of operation, has a direct influence on the equipment’s overall performance, safety, and reliability [1]. As bearings undergo long-term operation in mechanical systems, faults may occur in various parts, and changes in operating conditions often obscure fault features. Upon the occurrence of a fault, it may lead to substantial economic losses and can even be the cause of major accidents [2,3,4]. Therefore, to guarantee the proper functioning of machinery, reduce or even prevent faults, real-time monitoring of the equipment’s health condition and fault location diagnosis based on signal changes are crucial. Regular maintenance can help reduce potential failures, save maintenance costs, avoid major accidents, and promote sustainable development, all of which are of significant practical importance [5].

Traditional data-driven methods typically involve steps such as feature extraction, feature selection, and model training. The feature extraction phase processes raw vibration signals, including time–domain features, frequency–domain features, and time–frequency domain features [6,7]. The most representative features are selected from the original feature set using dimensionality reduction techniques (e.g., PCA) or correlation analysis for model training [8,9,10]. Traditional machine learning techniques, including random forest, support vector machine (SVM), and logistic regression, are then used to train classifiers to identify different fault modes [11,12]. Zhu et al. [13] developed an SVM-based fault diagnosis methodology and optimized the SVM using quantum genetic algorithms (QGA) to increase fault diagnosis precision and effectiveness in rotating machinery. Hong et al. [14] introduced a probabilistic classification model using an SVM classifier for the real-time identification of faults in power transformers. Deng et al. [15] introduced the EWTFSFD diagnosis approach, combining EWT, fuzzy entropy, and SVM, which performs fault recognition by decomposing signals, extracting features, and constructing classifiers. The experimental results confirmed its accuracy and effectiveness, with better signal decomposition using EWT. Xiao et al. [16] presented a rotating machinery fault diagnosis method, which combines improved variational mode decomposition (IVMD) with convolutional neural networks (CNN). They first solved the time–domain feature extraction problem using the improved VMD that automatically optimizes the number of modes. Then, they selected high-correlation components using correlation analysis to reconstruct the signal, and 2D time–frequency domain feature maps were extracted using CWT. This method is suitable for complex environments and achieves high recognition rates.

Deep learning techniques have apparent advantages in automatically extracting complex features and uncovering hidden information from data, compared to traditional machine learning methods. Their rapid development has gradually led to their application in the fields of Prognostics and Health Management (PHM) [17,18,19,20]. Wang et al. [21] proposed a fault diagnosis scheme based on Multi-scale Diversity Entropy (MDE) and Extreme Learning Machine (ELM), demonstrating that its classification accuracy in rotating machinery pattern recognition surpasses that of existing methods such as Sample Entropy, Fuzzy Entropy, and Permutation Entropy. He et al. [22] introduced a few-shot learning method based on a fine-tuned GPT-2 model for fault diagnosis of all-ceramic bearings, achieving high diagnostic accuracy with very few labeled samples. Tian et al. [23] studied an iron-core fluxgate sensor for motor condition monitoring, showing that, compared to coreless sensors, the iron-core sensor has a stronger and more sensitive measurement of electromotive force in noisy environments. Bai et al. [24] addressed the challenges of using deep learning for mechanical fault diagnosis, such as the dependence on large data, long training times, and performance degradation with inconsistent data, by introducing an innovative data representation method using the fractional order Fourier transform (FRFT) and recursive graph transformation for bearing fault diagnosis. Liu et al. [25] developed a rolling bearing fault diagnosis method using 1D and 2D CNNs for two-domain information learning. Tests on the CWRU and DUT datasets confirmed that the approach attains high recognition accuracy and operates effectively without manual expertise. The method also demonstrated its superiority and robustness in tests with strong noise vibration data. The fault diagnosis approaches referenced in the previous literature depend exclusively on data from a single sensor. However, in actual industrial production environments, there are various uncontrollable factors that cause noise in the signals. Therefore, the data gained from a single sensor is limited.

For the various information problems associated with single sensors, how to use multi-sensor data fusion to enhance fault diagnosis model accuracy has become a key research focus. Three fusion levels are distinguished: data-level fusion, feature-level fusion, and decision-level fusion [26]. Shao et al. [27] proposed a cross-domain fault diagnosis method for ceramic bearings based on meta-learning and multi-source heterogeneous data fusion. This method effectively addresses issues of data dimension differences and feature consistency, achieving an average diagnostic accuracy of 98.91% across six cross-domain scenarios, demonstrating excellent noise resistance. Dong et al. [28] introduced a lightweight convolutional dual-regularized contrastive transformer based on multi-sensor data fusion for small sample fault diagnosis of aerospace bearings. By integrating cliff entropy weighting and the Diwaveformer architecture, this method achieved efficient and accurate fault identification. Saucedo-Dorantes et al. [29] proposed a data-driven diagnostic method based on deep feature learning. Utilizing a deep autoencoder model and feature fusion techniques, this method effectively diagnosed and identified faults in metal, hybrid, and ceramic bearings within motor systems, and its adaptability and performance were validated across different motor systems. Chao et al. [30] introduced a multi-sensor fusion method utilizing a convolutional neural network receiving three-channel vibration data with decision-level fusion. The method was validated using an axial piston pump experiment, showing its effectiveness. Although the above deep learning-based multi-sensor fusion fault diagnosis methods have achieved good results, certain limitations still exist. First, the diagnostic results are not ideal when monitoring signals in real industrial environments encounter significant noise interference. The robustness of the above deep learning fusion methods to noise needs to be further enhanced. Second, most diagnostic methods use one or more vibration sensors, which require precise installation of contact-type sensors, but less attention is given to non-contact sensors like acoustic sensors and temperature sensors. The combination of multiple signals was demonstrated to improve the efficacy of fault diagnosis methods. Third, multi-sensors often provide heterogeneous data types, including images, time series, acoustic signals, etc. Each data type may differ in time and spatial scales. How to efficiently fuse these data from different scales and extract useful fault information remains a challenging problem.

To address the aforementioned issues and enhance the performance of multi-sensor acoustic-vibration fusion fault diagnosis methods, while comprehensively utilizing features at different levels and scales to capture multi-level detailed information, this paper introduces a bearing fault diagnosis approach using a multi-channel multi-scale spatiotemporal convolutional cross-attention fusion network. Specifically, CWT is employed to convert the 1D acoustic and vibration signals into 2D time–frequency images. After these images undergo rough feature extraction using ResNet, deep feature extraction is performed using the multi-scale temporal convolution module (MTCM) and multi-feature extraction block (MFE) to obtain time and spatial features. These features subsequently undergo processing using the dual cross-attention (DCA) mechanism, where multi-scale channel and spatial cross-fusion of the features is performed to achieve adaptive fusion of global and local features. Finally, the fused features are classified and recognized in vector form using GAP, FC, and softmax. The primary contributions of this work include the following:

(1) A bearing fault diagnosis framework based on a multi-channel multi-scale spatiotemporal convolutional cross-attention fusion network (MMSTCCAFN) is proposed. This framework not only eliminates the requirement for manual feature extraction in conventional diagnostic approaches but also addresses the limitations of using a single sensor. At the same time, it effectively realizes the complementary fusion of spatiotemporal feature information from multi-sensor acoustic and vibration signals.

(2) The feature extraction module, MTCM, is improved. Building upon the original TCM, the expansion of the inception layer is enhanced by assigning weights using the softmax function, and the weighted sum is improved to emphasize important features while suppressing less significant information.

(3) A multi-scale spatial feature extraction module (MFE) is introduced. This module employs convolution and attention operations, making it suitable for processing images of different scale sizes. It effectively captures and integrates both local and global contextual information from the images.

(4) A dual cross-attention module (DCA) is introduced, which includes channel cross-attention (CCA) and spatial cross-attention (SCA) mechanisms. This module adaptively captures multi-scale channel and spatial dependencies, enabling the learning of information at various spatial scales.

The organization of the article is as follows. Section 2 briefly introduces the continuous wavelet transform and the attention mechanism. Section 3 details the proposed method, including MTCM, MFE, DCA, and MMSTCCAFN, along with the bearing fault diagnosis framework based on MMSTCCAFN. Section 4 validates the efficacy of the proposed methodology using two experimental cases. And Section 5 presents the conclusion.

## 2. Related Work

### 2.1. Continuous Wavelet Transform

Wavelet transform, as a time–frequency analysis technique for signals, uses wavelet bases to extract information from both the time and frequency domains and is extensively applied in fault diagnosis. Continuous Wavelet Transform (CWT) is a typical wavelet transform method. Compared to Short-Time Fourier Transform (STFT), CWT offers a better balance of time and frequency resolution. Compared with the Discrete Wavelet Transform (DWT), CWT responds quickly and contains rich information, making it more advantageous for the recognition and analysis of similar signals. For a function $f(t)\in L^{2}(\mathbb{R})$, the formula for CWT of the function is as follows [31]:

(1) $$CWT(a,b)=[f(t),\varphi_{a,b}(t)]=\frac{1}{\sqrt{a}}\int_{-\infty }^{+\infty }f(t)\varphi^{*}(\frac{t-b}{a})dt$$

where f(t) is the original signal, b is the shift factor, φ is the mother wavelet or basic wavelet function, and a is the scale factor.

This study selected the Morlet wavelet as the mother wavelet function, which is expressed as follows:

(2) $$\varphi_{\mathrm{morlet}}(t)=\pi^{-\frac{1}{4}}e^{i\omega_{0}t}e^{-\frac{t^{2}}{2}}$$

By substituting the Morlet wavelet into the continuous wavelet transform formula, the expression for the Morlet wavelet transform is obtained:

(3) $$W_{\mathrm{morlet}}(a,b)=\pi^{-\frac{1}{4}}\frac{1}{\sqrt{a}}\int_{-\infty }^{\infty }f(t)e^{-\frac{{\left(t-b\right)}^{2}}{2a^{2}}}e^{-i\frac{\omega_{0}}{a}(t-b)}dt$$

In the above expression, $e^{-\frac{{\left(t-b\right)}^{2}}{2a^{2}}}$ represents the Gaussian function, and $e^{-i\frac{\omega_{0}}{a}(t-b)}$ is the complex exponential function, which characterizes the frequency oscillation properties of the signal on the time axis.

The Morlet wavelet, due to its superior time–frequency localization properties, can provide excellent time and frequency resolution simultaneously. This makes it ideal for processing signals that contain rich high-frequency components and transient characteristics. Particularly in mechanical fault diagnosis, it can effectively capture high-frequency components and sudden fault signals. In this manner, CWT can more accurately extract multi-scale, multi-resolution features, thereby enhancing the subsequent fault diagnosis performance.

### 2.2. Attention Mechanism

Bahdanau et al. [32] first introduced the attention mechanism in 2014 for machine translation tasks. The Google team [33] proposed the Transformer, which directly used the self-attention mechanism to process the entire input sequence. Initially, the attention mechanism was mainly used in Natural Language Processing (NLP) tasks, was subsequently adapted for computer vision. In recent years, it has also started to be applied in fault diagnosis [34,35]. In traditional sequence models like Recurrent Neural Networks (RNN) and Long Short-Term Memory Networks (LSTM), as the sequence length increases, the model tends to lose information from earlier inputs, thus facing challenges in capturing long-range dependencies. However, the attention mechanism establishes direct connections between various positions in the sequence, effectively capturing dependencies between them, regardless of their distance. The attention mechanism can concentrate on the most critical information for the present assignment within an extensive volume of input data, diminishing the attention given to irrelevant information and addressing the issue of information overload, thereby improving task efficiency and accuracy.

In the attention mechanism, Q (Query), K (Key), and V (Value) are mapped to corresponding vectors using mapping matrices. The similarity between Q and K:

(4) $$\mathrm{Attention}(Q,K,V)=\mathrm{softmax}\left(\frac{QK^{T}}{\sqrt{d_{k}}}\right)V$$

where $QK^{T}$ represents the similarity, d is the dimension of the key vector, and $d_{k}$ is the dimensionality of K.

## 3. Proposed Method

### 3.1. Multi-Scale Temporal Convolution Module

Vibration signals are typically represented as time series data, characterized by significant variations over the time dimension. In time series with rich and diverse temporal features, temporal convolution can capture features within specific time ranges to some extent. However, due to the relatively simple structure of the model, it is only suitable for time series data with relatively uniform features. In bearing fault diagnosis, it is crucial to comprehensively capture the various features within the time series data. Multi-scale temporal convolution leverages convolution kernels for feature extraction across the temporal dimension. Dilated convolution networks increase the depth of the layers, allowing for exponentially large receptive fields, thus effectively handling long sequences. The receptive field size of the computational model can be calculated [36]. Assuming an initial dilation factor of 1, the receptive field size of a k-layer dilated convolution network with a kernel size of r is given using the following formula:

(5) $$R=\left\{\begin{array}{ll} k(r-1)+1, & q=1\\ \frac{\left(r-1)(q^{k}-1\right)}{\left(q-1\right)}+1, & q>1 \end{array}\right.$$

**Figure 1:**
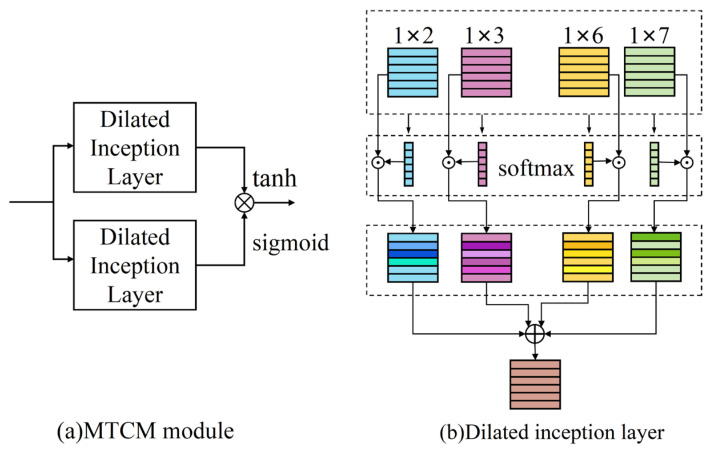
The structure of the multi-scale temporal convolution module: (**a**) MTCM module; (**b**) Dilated inception layer.

As shown in Figure 1, the MTCM module comprises two dilated inception layers (DIL). One DIL applies a tanh activation function and acts as a filter. The other DIL, which uses a sigmoid activation function, serves as a gate for information flow control to subsequent modules. This mechanism was proven to have a significant impact on the flow of information across layers in temporal convolution networks. The dilated convolution represented by $X⋆f_{1\times r}$ is expressed as:

(6) $$X⋆f_{1\times r}(t)=\sum_{s=0}^{r-1}f_{1\times r}(s)X(t-q\times s)$$

where q represents the dilation factor. Extracting time patterns over different ranges from the time series data is achieved using the concurrent deployment of four filters with dimensions 1 × 2, 1 × 3, 1 × 6, and 1 × 7. Afterward, concatenation of global features across *N* scales along the second dimension, resulting in $X^{\prime }\in {\mathbb{R}}^{C\times N\times 1}$. Then, the respective weight representations are obtained along the second dimension (i.e., the *N*-dimension) using the softmax function:

(7) $$\begin{array}{l} X^{\prime }=\mathrm{concat}(X⋆f_{1\times 2},X⋆f_{1\times 3},X⋆f_{1\times 6},X⋆f_{1\times 7})\\ W\in {\mathbb{R}}^{C\times N\times 1}=\mathrm{softmax}(X^{\prime }) \end{array}$$

By summing the four multi-scale feature values along the channel dimension, the number of channels is restored to be the same as the input. Subsequently, the two dilated inception layers, after being processed by two different activation functions, undergo element-wise multiplication as follows:

(8) $$\hat{X}=\tanh({X^{\prime }}_{1})⊙\mathrm{sigmoid}({X^{\prime }}_{2})$$

where ${X^{\prime }}_{1}$ and ${X^{\prime }}_{2}$ are the parameters of the two dilated inception layers.

Overall, through the use of multi-scale temporal convolution and the tanh and sigmoid activation functions, the module gains the ability to capture long-term, multi-scale temporal dependencies. Additionally, it enhances the extraction and selection of features.

### 3.2. Multi-Feature Extraction Block

**Figure 2:**
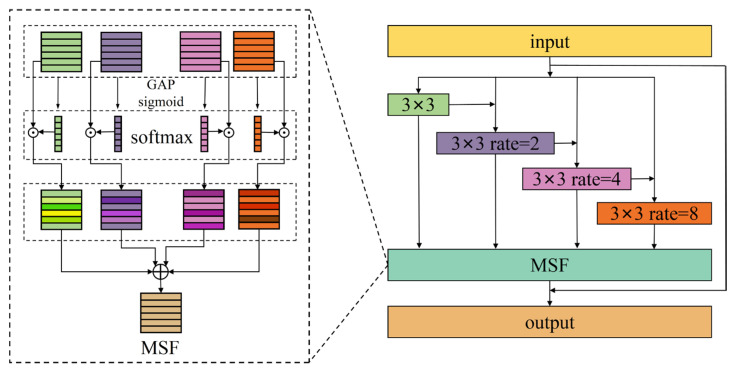
The structure of the multi-feature extraction block.

As noted by MDE [21], this method has the advantages of high consistency, computational efficiency, and strong robustness. However, in the feature extraction process across different frequency ranges, it primarily considers the low-frequency part, which contains less fault information, while neglecting the useful fault information in the high-frequency part. To better understand the spectral structure of acoustic signals and comprehensively capture both low-frequency and high-frequency components of the signal, this study introduces an MFE block [37]. This block performs multi-scale feature extraction and fusion on the acoustic signals to enhance the image information. The MFE architecture is illustrated in Figure 2. The input feature X is passed through four 3 × 3 convolution layers with different dilation rates to capture multi-scale image features:

(9) Yi=Conv3×3rate=(X)1i=1Conv3×3rate=(X+Yi−1)2(i−1)1<i≤n

where n is the number of irregular convolutions. This module increases network width while maintaining depth, enabling it to simultaneously extract both local and global information.

After the feature extraction, MSF, a new fusion method, is proposed to fuse features from all different scales. First, global feature representation is obtained via performing global average pooling (GAP) along the spatial dimension, followed by 1 × 1 convolution to model the inter-channel correlations, and channel descriptors are generated using a sigmoid activation function:

(10) $$X_{0}^{\prime }\in {\mathbb{R}}^{C\times 1\times 1}=\mathrm{sigmoid}\left(Conv\left(\mathrm{AvgPool}\left(X_{0}\right)\right)\right)$$

Subsequently, *N* scales of global features undergo second-dimension concatenation to obtain $X\_\in {\mathbb{R}}^{C\times N\times 1}$, and the respective weight representations are obtained along the second dimension (i.e., the *N*-dimension) using the softmax function:

(11) $$\begin{array}{l} X\_\;=\mathrm{Concat}({X^{\prime }}_{0},{X^{\prime }}_{1},\ldots ,{X^{\prime }}_{N})\\ W\in {\mathbb{R}}^{C\times N\times 1}=\mathrm{softmax}(X\_)\\ W[:,i,:]\in {\mathbb{R}}^{C\times 1\times 1} \end{array}$$

The generated weights are then element-wise multiplied with the corresponding inputs and summed to produce $Y\in {\mathbb{R}}^{C\times H\times W}$, representing the fused feature representation.

(12) $$Y=W[:,0,:]⊙X_{0}+W[:,1,:]⊙X_{1}+\ldots +W[:,N,:]⊙X_{N}$$

The MFE module captures both local and global image context by processing features from different scales and applying convolution and weighting operations to fuse them effectively. This enables the effective identification of subtle anomalies and differences between various fault modes in the operation of bearings.

### 3.3. Dual Cross-Attention Module

#### 3.3.1. Multi-Scale Feature Embedding Module

The Multi-Scale Feature Embedding Module extracts feature maps from n encoders and uses the generated feature maps as input. The output feature map $E_{i}$ of each encoder at different scales has dimensions ${\mathbb{R}}^{C_{i}\times \frac{H}{2^{i-1}}\times \frac{W}{2^{i-1}}}$, where i=1,2,…n. $P_{i}^{s}=\frac{P^{s}}{2^{i-1}}$ represents the block size. The feature map undergoes average pooling, using a pooling kernel size and stride both equal to $P_{i}^{s}$. This average pooling method flattens the feature maps into a 2D sequence, followed by a 1 × 1 depth wise separable convolution for feature mapping, ensuring consistency in the dimension across different scales and preserving the original number of channels [38]. The formula can be expressed as:

(13) $$T_{i}=\mathrm{DConv}1D_{E_{i}}\left(\mathrm{Reshape}\left(\mathrm{AvgPool}2D_{E_{i}}\left(\;E_{i}\right)\right)\right)$$

where $T_{i}\in {\mathbb{R}}^{P\times C_{i}}\;(i=1,2,\ldots ,n)$ represents the feature map after flattening in the i-th encoder stage. It is worth noting that each $T_{i}$ for different scales is the same, making it easier for subsequent processing.

#### 3.3.2. Channel Cross-Attention (CCA)

**Figure 3:**
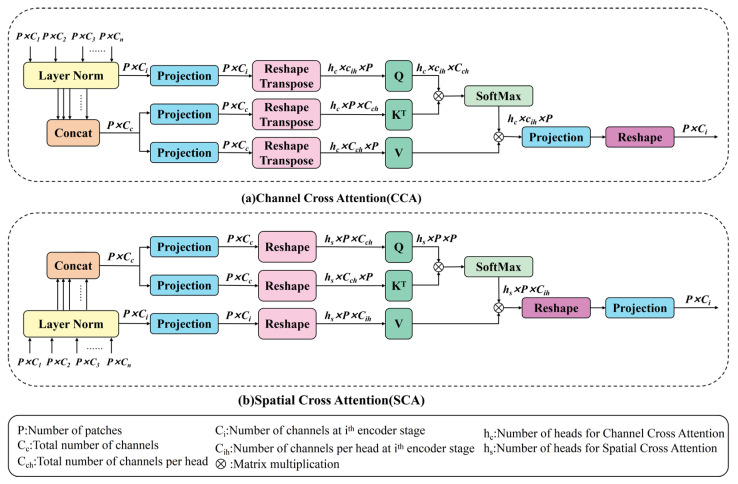
The structure of the dual cross-attention module is composed of two modules: (**a**) Channel Cross-Attention (CCA); (**b**) Spatial Cross-Attention (SCA).

As shown in Figure 3a, the CCA module takes the multi-scale feature embedding module’s generated tokens $T_{i}$ as input. First, layer normalization is applied to each layer. Then, the tokens $T_{i}(i=1,2,\ldots n)$ are concatenated along the channel dimension, and 1 × 1 depth wise convolution projection is applied to form the keys and values $T_{c}$, while $T_{i}$ is used as the query [38]. The formula is as follows:

(14) $$\begin{array}{l} Q_{i}={\mathrm{DConvD}}_{Q_{i}}(T_{i})\\ K={\mathrm{DConvD}}_{K}(T_{c})\\ V={\mathrm{DConvD}}_{V}(T_{c}) \end{array}$$

where $Q_{i}\in {\mathbb{R}}^{P\times C_{i}},K\in {\mathbb{R}}^{P\times Cc},V\in {\mathbb{R}}^{P\times Cc}$ represent the projected query, key, and value, respectively. Then, matrix multiplication is performed between the query and key to obtain the relevance matrix. Lastly, softmax normalization is performed on the relevance matrix. The formula for CCA is as follows:

(15) $$\mathrm{CCA}(Q_{i},K,V)=\mathrm{Softmax}\left(\frac{Q_{i}^{T}K}{\sqrt{C_{c}}}\right)V^{T}$$

where $Q_{i}$, K and V represent the query, key, and value, respectively, and $\frac{1}{\sqrt{C_{c}}}$ acts as the scaling factor. Lastly, the computed attention weights subsequently generate a weighted summation of the values.

#### 3.3.3. Spatial Cross-Attention (SCA)

As presented in Figure 3b, the SCA module first applies layer normalization and concatenation across the channel dimension. Then, 1 × 1 depth convolution projections generate the query, key, and value. The formula is as follows [38]:

(16) $$\begin{array}{l} Q=\mathrm{DConv}1D_{Q}({\bar{T}}_{c})\\ K={\mathrm{DConv}1D}_{K}({\bar{T}}_{c})\\ Vi={\mathrm{DConv}1D}_{V_{i}}({\bar{T}}_{i}) \end{array}$$

where $Q\in {\mathbb{R}}^{P\times Cc},\;K\in {\mathbb{R}}^{P\times Cc}$, and $V_{i}\in {\mathbb{R}}^{P\times Cc}$ represent the projected query, key, and value, respectively. Therefore, the formula for SCA is:

(17) $$\mathrm{SCA}(Q,K,V_{i})=\mathrm{Softmax}\left(\frac{QK^{T}}{\sqrt{d_{k}}}\right)V_{i}$$

where Q, K and $V_{i}$ are the embedding matrices of the query, key, and value, and $\frac{1}{\sqrt{d_{k}}}$ serves as the scaling factor. In a multi-head configuration, $d_{k}=\frac{c_{c}}{h_{c}}$, where $h_{c}$ represents the number of heads. The calculated attention weights enable weighted aggregation of the value matrix.

We employ a dual cross-attention module to receive the acoustic and vibration time–frequency feature maps after feature extraction. These feature maps are then passed to the channel and spatial attention modules for feature fusion. The channel and spatial attention modules capture long-range dependencies in the channel and spatial dimensions, respectively. The cross-attention mechanism makes these two complementary to each other, allowing for a more comprehensive detection of different types of faults. The complementarity of different signal sources helps provide more complete fault information.

Specifically, the SCA and CCA modules within the DCA module are cascaded. The feature maps first pass through the CCA module, and the resulting output features are then passed to the SCA module. Subsequently, the output features from the SCA and CCA modules are concatenated along the channel dimension:

(18) $$Y_{\mathrm{combined}}=\mathrm{Concat}\left(Y_{CCA},Y_{SCA}\right)$$

The combined features, after further processing, effectively integrate the characteristics of both attention mechanisms, thereby enhancing the quality of the final feature representation. This dual cross-attention mechanism effectively integrates the low-level multi-scale encoder features and captures detailed representations, narrowing the semantic gap. Therefore, it can better capture critical bearing fault information and improve fault diagnosis results.

### 3.4. Multi-Channel Multi-Scale Spatiotemporal Convolutional Cross-Attention Fusion Network

**Figure 4:**
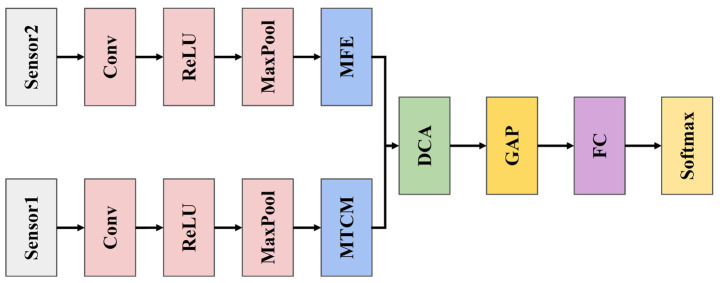
The structure of MMSTCCAFN.

To achieve diversity in multi-sensor data, a Multi-channel Multi-scale Spatiotemporal Convolutional Cross-Attention Fusion Network (MMSTCCAFN) is proposed, illustrated in Figure 4. This network adopts a parallel fusion structure. The time–frequency representations of acoustic and vibration signals first pass through initial convolution layers (Conv), activation functions (ReLU), and max-pooling layers (MaxPool) to extract primary features, reduce data dimensions, and decrease computational complexity. This process aids in capturing the fundamental characteristics of bearings under normal operation and various fault conditions. Next, the vibration time–frequency representations, after preliminary feature extraction, enter the MTCM. This module extracts multi-scale features from time series data using convolution kernels of different sizes and hierarchical structures, capturing information across various time scales. Concurrently, the acoustic time–frequency representations enter the MFE module to further extract multi-scale spatial features, enhancing the richness and accuracy of feature representations. The two modules complement each other, enabling the capture of multi-scale, detailed features of bearings under different fault types. Subsequently, the two parallel multi-scale feature branches are fed into the DCA module for feature fusion, effectively integrating features from different sensors and enhancing the overall diversity of feature representations. Finally, the fused features undergo a Global Average Pooling layer (GAP) to further reduce dimensions while retaining important information, and the fault types of the bearings are output through a Fully Connected layer (FC) and a softmax layer. Through the design of these two branches, MMSTCCAFN efficiently extracts and fuses features from various sensor data types, enhancing model classification performance.

### 3.5. Fault Diagnosis Framework Based on MMSTCCAFN

**Figure 5:**
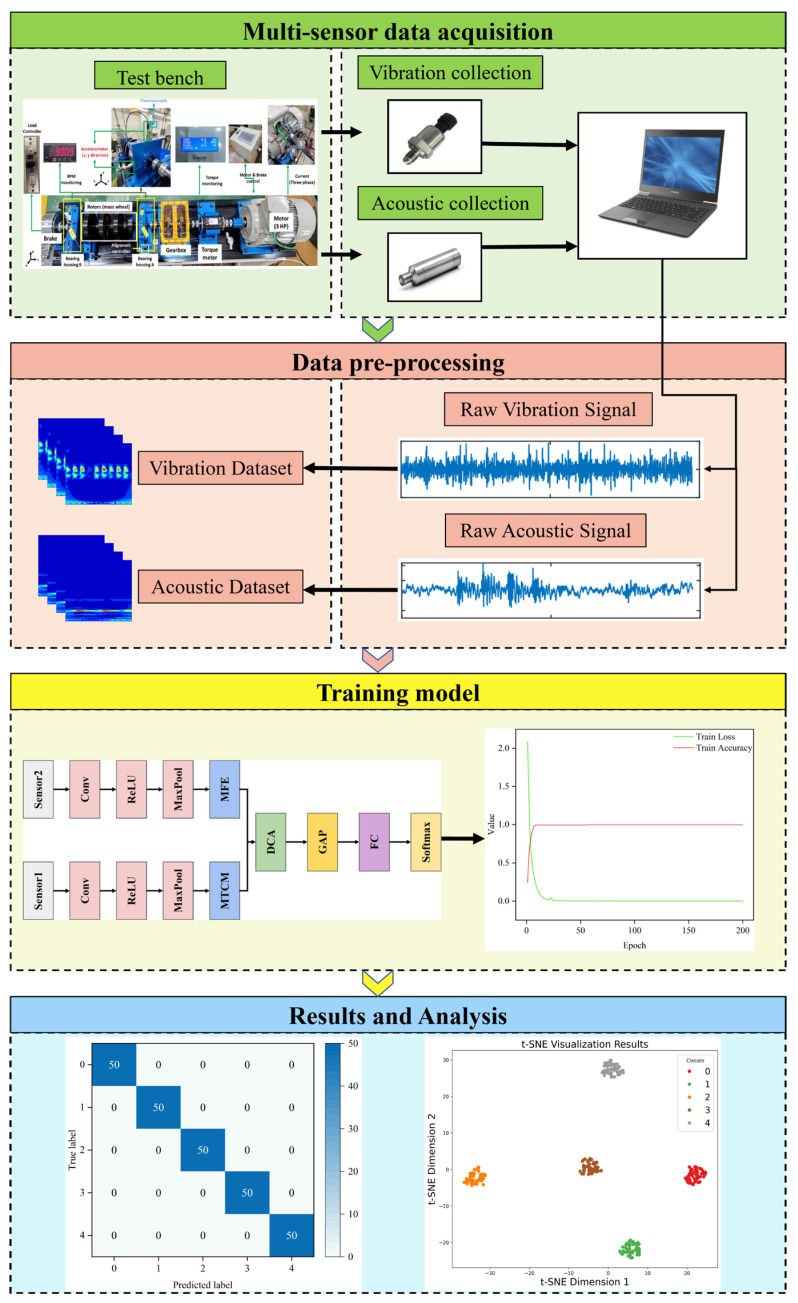
The proposed framework’s diagnostic process.

A bearing fault diagnosis framework utilizing MMSTCCAFN is proposed, as illustrated in Figure 5. The specific steps are as follows:

Step 1: Multi-sensor data collection. Vibration and acoustic sensors are installed on and around the bearing surface to collect vibration and acoustic signals under healthy and defective bearing states.

Step 2: Data preprocessing of the collected signals. CWT is applied to generate time–frequency maps, and the dataset is divided into training, validation, and test sets.

Step 3: Training MMSTCCAFN using the training set. The MMSTCCAFN architecture is initialized, and during training, the Adam optimizer is applied to adjust the weights, promoting gradual loss decrease and continuous feature extraction.

Step 4: After training is complete, the trained MMSTCCAFN model needs to be tested and evaluated. The model’s generalization ability is evaluated using the test set, establishing the fault diagnosis performance of the framework.

## 4. Experimental Validation

### 4.1. Case 1: University of Ottawa Rolling-Element Dataset

#### 4.1.1. Introduction to the Dataset for Case 1

**Figure 6:**
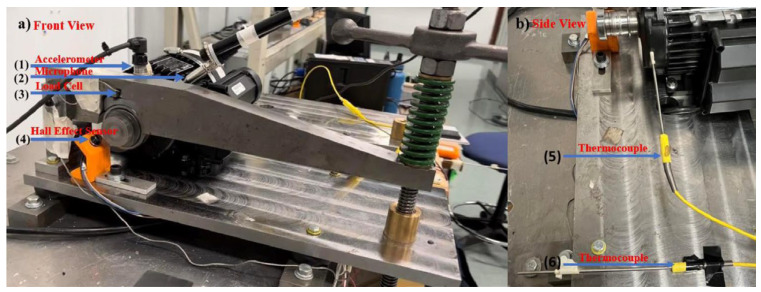
Test bench: (**a**) front view; (**b**) side view.

**Figure 7:**
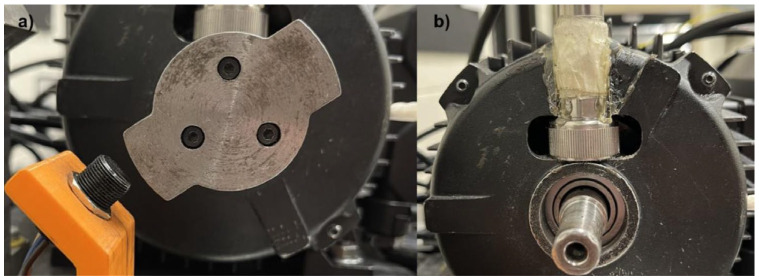
Close-up view: (**a**) two-toothed gear; (**b**) accelerometer placement.

**Figure 8:**
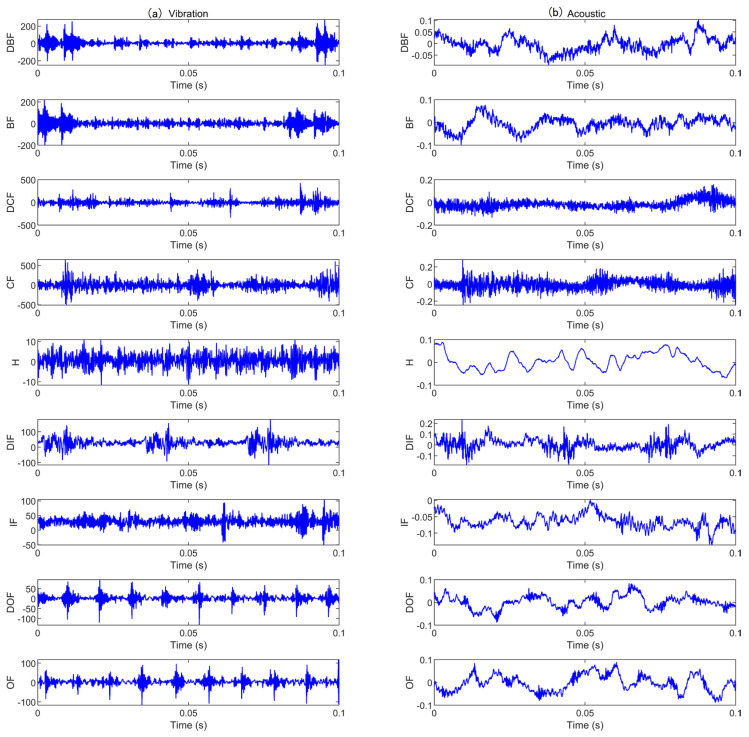
Original signals for various faults in case 1: (**a**) vibration signal; (**b**) acoustic signal.

We use the bearing vibration and acoustic fault signature datasets from the University of Ottawa, Canada. The data was collected using the (UORED-VAFCLS) test bench [39]. The experimental arrangement is illustrated in Figure 6 and Figure 7. A single-phase motor installed on a rigid plate constitutes the test bench, with the rigid plate supported by vibration isolation brackets. The motor shaft is sustained by two bearings, with the drive-end bearing being FAFNIR 203KD and the non-drive-end bearing being NSK 6203ZZ. The data acquisition equipment includes an NI-USB-6212, with an accelerometer (PCB, model 623C01) utilizing acquisition vibration signals and a microphone (PCB, model 130F20) used to collect acoustic signals. During data collection, the sampling frequency was 42 kHz, the load was 400 N, and the motor speed was 1750 rpm. Vibration and acoustic data were sequentially collected under nine different states, which are categorized into three groups: healthy, fault development, and fault. Groups fault development and fault include four different fault locations: inner race, outer race, ball, and cage, yielding nine total bearing operating conditions. The raw vibration and acoustic signals for the nine states are shown in Figure 8. Additionally, each operating condition consisted of 350 training samples and 50 testing samples. Table 1 provides comprehensive details regarding the sampling data for each condition.

#### 4.1.2. Effectiveness of the Proposed Method in Case 1

**Figure 9:**
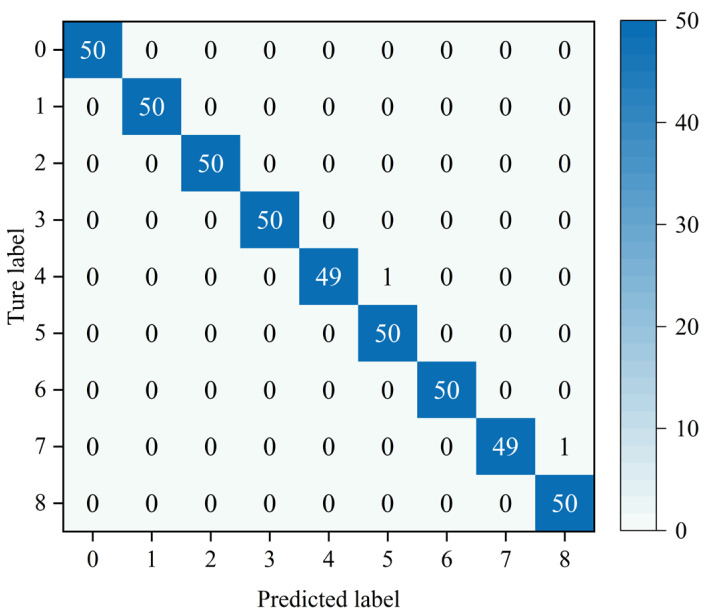
Confusion matrix of our proposed method in case 1.

**Figure 10:**
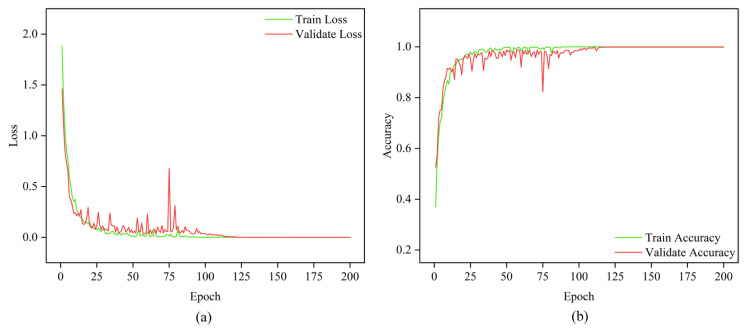
Iteration curve in case 1: (**a**) loss; (**b**) accuracy.

**Figure 11:**
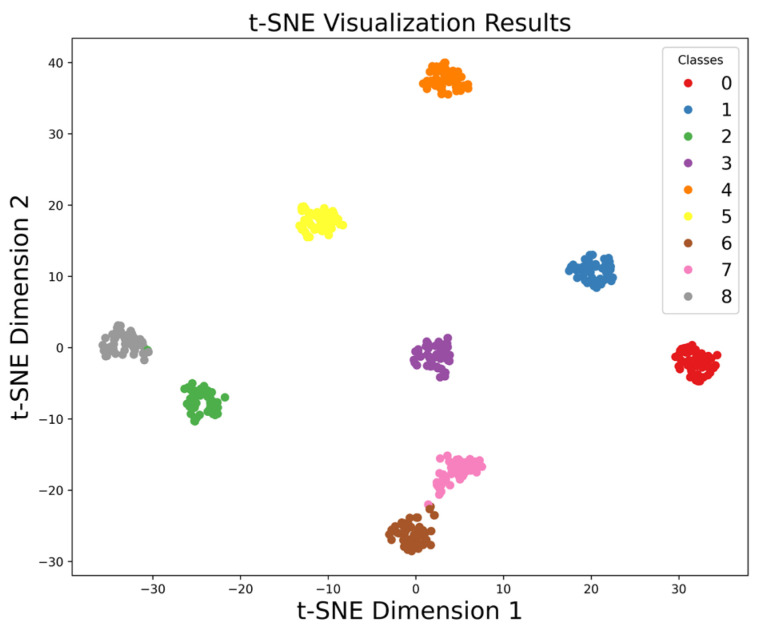
t-SNE visualization results of our proposed method in case 1.

The MMSTCCAFN hyperparameter settings are detailed in Table 2. To achieve optimal performance for the model, hyperparameters were selected and optimized as follows: (1) A smaller batch size enhances model generalization capability and convergence speed, but setting it too small may lead to excessive gradient noise. After multiple trials, a batch size of 32 was found to be a good choice, balancing training speed and model stability. (2) The number of training epochs was determined by observing the loss and accuracy curves. Experiments showed that after approximately 200 epochs, the training and validation losses stabilized, and the validation accuracy reached its peak. Therefore, 200 epochs were chosen as an effective balance between training time and model performance. (3) ReLU was chosen as the activation function due to its simplicity and computational efficiency, which are widely appreciated in deep learning. It helps mitigate the vanishing gradient issues and accelerates convergence speed during training. (4) The Adam optimizer integrates the advantages of momentum and adaptive learning rate optimization algorithms, effectively handling sparse gradients and being well-suited for models with large datasets and parameters. Multiple experiments confirmed that the Adam optimizer delivered the best performance in our model. (5) Learning rate selection governs weight adjustment magnitude during updates. A learning rate set too high may hinder convergence, while a learning rate set too low can slow down the training process excessively. Using multiple training iterations, a learning rate of 0.001 was proven to enable stable model convergence within a reasonable timeframe and achieve high accuracy. The model is first trained on the training set and then saved. The saved model is then used to perform fault diagnosis on the test set. Finally, the fault diagnosis performance of MMSTCCAFN is evaluated using a confusion matrix, iteration curves, and t-SNE visualization techniques. Figure 9 presents the confusion matrix for the test samples. Figure 10 presents the Loss Curve and Accuracy Curve after 200 iterations. As the iteration count increases, both training and validation loss curves decrease and eventually level off. Similarly, the training accuracy curve and validation accuracy curve gradually increase with the number of iterations and ultimately stabilize. Using t-SNE visualization, we can observe that the data points of different classes form distinct clusters. Figure 11 shows that the distribution of data points across nine different categories is visually clear, demonstrating the method’s effectiveness in fault classification.

**Figure 12:**
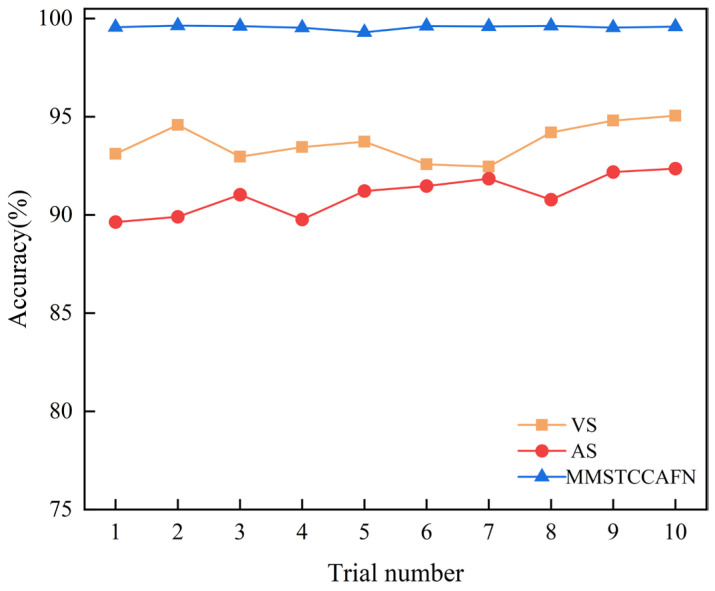
Comparison of inputs from different sensors in case 1.

Additionally, to discuss the effectiveness of MMSTCCAFN and demonstrate multi-sensor fusion diagnostics, we compared it with diagnostic methods based on single sensors. Two different input methods were used: a single vibration sensor (VS) and a single acoustic sensor (AS). In the VS method, the MTCM branch of MMSTCCAFN is executed, followed directly by GAP, FC, and softmax. Similarly, in the AS method, the MFE branch of MMSTCCAFN is executed, followed directly by GAP, FC, and softmax to output the diagnostic results. Each method was repeated 10 times, with statistical results for each sensor shown in Table 3 and detailed results in Figure 12. The results indicate that the diagnostic accuracy using single-input signals from VS and AS is lower than that of MMSTCCAFN. The non-linear behavior of vibration sensor data may result in complex features in the vibration signal. Additionally, vibration signals are prone to external environmental noise during transmission, which reduces signal clarity and complicates fault diagnosis. The attenuation and deformation of acoustic signals in the medium can affect the accuracy of the acoustic wave signals, making source localization more challenging. Acoustic sensors are also easily influenced by environmental changes, leading to signal distortion or attenuation. However, MMSTCCAFN integrates signals from both sensors, leveraging their respective advantages while compensating for their shortcomings. Firstly, vibration and acoustic signals each have different sensitive frequency bands and characteristics in mechanical fault detection, and their fusion can enhance the comprehensiveness of fault feature capture. Secondly, multi-sensor fusion can effectively reduce environmental noise interference from a single signal source, improving diagnostic accuracy and robustness. Additionally, the combination of vibration and acoustic signals provides complementary spatiotemporal characteristics, further improving the sensitivity and precision of fault detection. By combining the two types of signals, MMSTCCAFN achieves an average diagnostic accuracy of 99.56% with a low standard deviation of 0.0993, overcoming individual limitations and enhancing the accuracy and effectiveness of fault diagnosis.

#### 4.1.3. Ablation Experiments for Case 1

**Figure 13:**
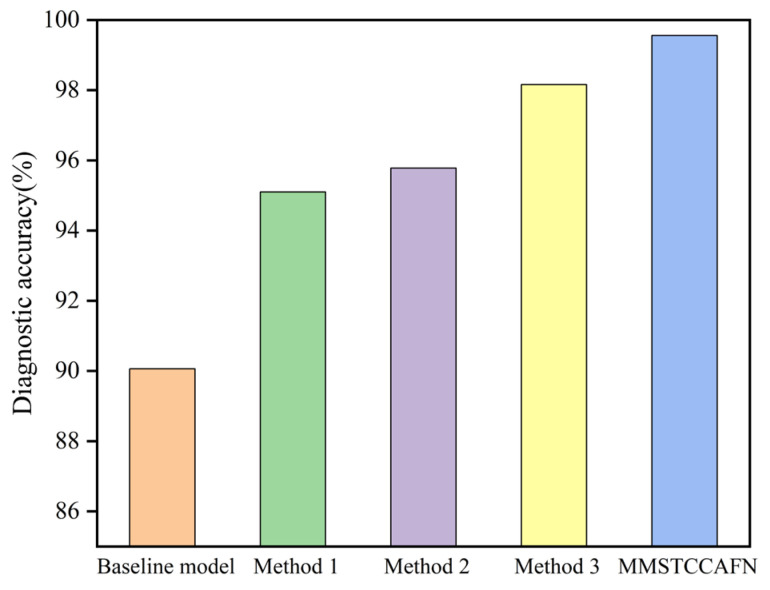
Ablation experiments in case 1: comparison of the diagnostic accuracy of different methods.

To thoroughly evaluate individual modules on MMSTCCAFN performance and to demonstrate the rationality of the proposed fusion framework, ablation experiments were performed using the dataset. (1) The baseline model consists of ResNet and sum fusion; (2) To validate the ability of MTCM in extracting multi-scale vibration time–frequency features and capturing sequence features across different time ranges, MTCM was removed from MMSTCCAFN, defined as Method 1; (3) To validate the ability of MFE in extracting local and global features from acoustic time–frequency map, MFE was similarly removed from MMSTCCAFN, defined as Method 2; (4) To validate DCA’s ability to capture complementary information during channel and spatial cross-attention feature fusion, sum fusion was used as a substitute for DCA, defined as Method 3. The structures of different methods are shown in Table 4, whereas the diagnostic accuracy outcomes from ablation experiments are shown in Figure 13 and Table 5. The results from the ablation experiments clearly show that MTCM improves accuracy. Compared to MMSTCCAFN, Method 1 increased accuracy by 4.46% and reduced the standard deviation by 0.53, indicating that the use of MTCM improves the model’s ability to extract vibration time–frequency features and enhances model stability. Compared to MMSTCCAFN, Method 2 increased accuracy by 3.78%, which is mainly due to the introduction of MFE, making the model more effective in extracting local and global image context information when processing images of different scales. MMSTCCAFN outperforms Method 3 by 1.4% in accuracy, demonstrating that DCA can adaptively capture multi-scale channel and spatial dependencies and better integrate information from different spatial scales, thus proving the efficacy and validity of multi-sensor acoustic and vibration signal fusion. Additionally, Method 1, Method 2, Method 3, and MMSTCCAFN exceed the baseline model’s peak performance while exhibiting reduced standard deviations, providing additional validation of the three modules’ efficacy. Overall, the proposed approach demonstrates outstanding diagnostic performance, achieving the highest accuracy of 99.64% compared to other approaches.

#### 4.1.4. Comparison with Other Methods in Case 1

**Figure 14:**
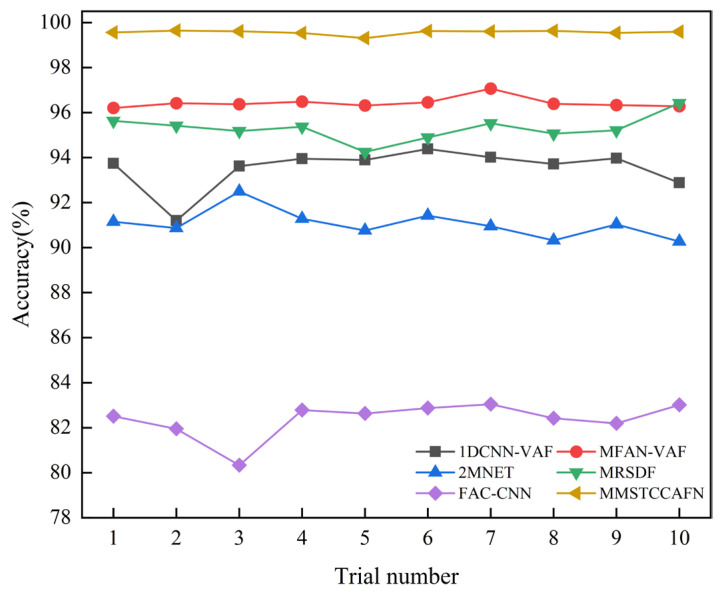
The comparison results of different architectures in case 1.

To further validate the advantages of MMSTCCAFN, five fusion methods are compared: 1DCNN-VAF [40], MFAN-VAF [41], 2MNET [42], MRSDF [43], and FAC-CNN [44]. 1DCNN-VAF: This approach extracts features from raw vibration and acoustic signals and then fuses them at the feature level using a 1D-CNN network. MFAN-VAF: This is an adversarial model for deep multi-sensor vibration and acoustic signal fusion based on transfer learning. It includes a multi-signal fusion module and an embedded residual network, aiming to achieve cross-domain fault diagnosis. 2MNET: This is a multi-sensor, multi-scale model that fuses multi-directional vibration signals using correlation kurtosis weighting. It performs fault diagnosis by extracting multi-scale features using an optimized network and combining them using the pyramid principle for feature fusion. MRSDF: This is a novel multi-rate sampling data fusion fault diagnosis method that uses CNN to automatically learn raw data features, and LSTM to mine time correlations and encode temporal information. FAC-CNN: The atrous convolution mechanism enables this CNN to adaptively fuse multi-source data, aiming to mine feature information from raw signals and avoid complex feature engineering. The MMSTCCAFN training protocol established a batch size of 32, the initial learning rate was set to 0.001, and the maximum number of iterations was set to 200. The parameter configuration of MMSTCCAFN can be found in Table 2, while the parameters for the other comparison methods are provided in the corresponding references. Each method was tested 10 times to ensure fairness in diagnostic results. Table 6 shows the statistical outcomes of the 10 trials for each method, with detailed results shown in Figure 14. The average accuracy of the five comparison methods (1DCNN-VAF, MFAN-VAF, 2MNET, MRSDF, FAC-CNN) is 93.54%, 96.43%, 91.05%, 95.29%, and 82.37%, respectively. Due to the multi-channel and multi-scale feature cross-fusion, MMSTCCAFN, which achieves an average accuracy of 99.56%, is able to better learn important information from the acoustic and vibration signals. Compared to the five comparison approaches, MMSTCCAFN demonstrates superior diagnostic performance. Furthermore, MMSTCCAFN indicates excellent stability in fault diagnosis with a standard deviation of 0.0993, lower than the other five methods. Notably, the highest accuracy of MMSTCCAFN is 99.64%, and the lowest is 99.30%, still outperforming all comparison methods and fully validating its superiority.

**Figure 15:**
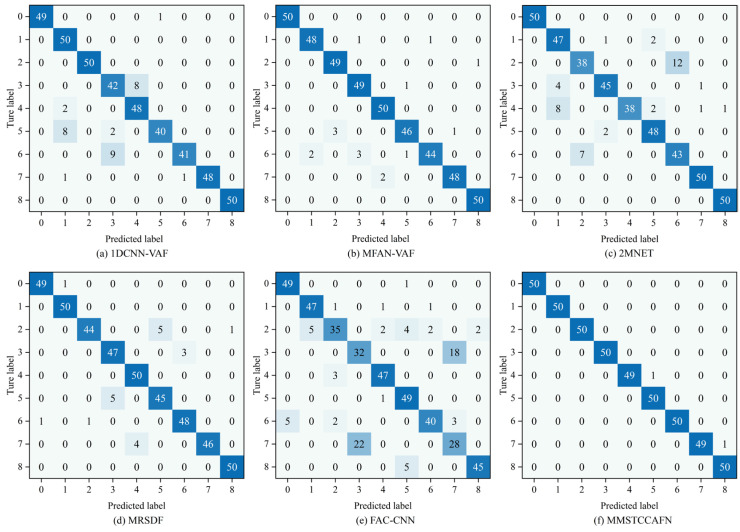
Confusion matrix of different methods in case 1: (**a**) 1DCNN-VAF; (**b**) MFAN-VAF; (**c**) 2MNET; (**d**) MRSDF; (**e**) FAC-CNN; (**f**) MMSTCCAFN.

Additionally, the diagnostic performance of the aforementioned methods for nine different bearing conditions was analyzed using confusion matrices. Figure 15 presents the confusion matrices for the different methods. For FAC-CNN and 2MNET, a significant number of samples were misclassified into incorrect categories, indicating substantial overlap when identifying different bearing conditions. Among the six comparative approaches, MFAN-VAF and MRSDF demonstrated better differentiation capabilities. Satisfactorily, MMSTCCAFN was able to effectively distinguish the nine bearing conditions with almost no misclassifications, further proving the superiority of MMSTCCAFN.

### 4.2. Case 2: Bearing Fault Dataset from the Korea Advanced Institute of Science and Technology

#### 4.2.1. Introduction to the Dataset for Case 2

**Figure 16:**
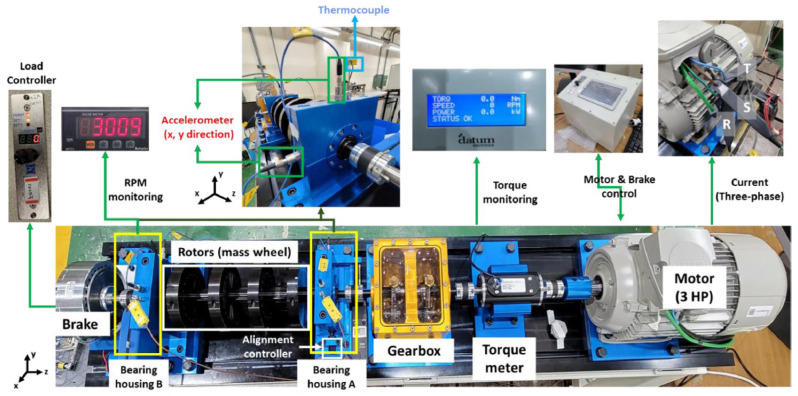
Test bench in case 2.

**Figure 17:**
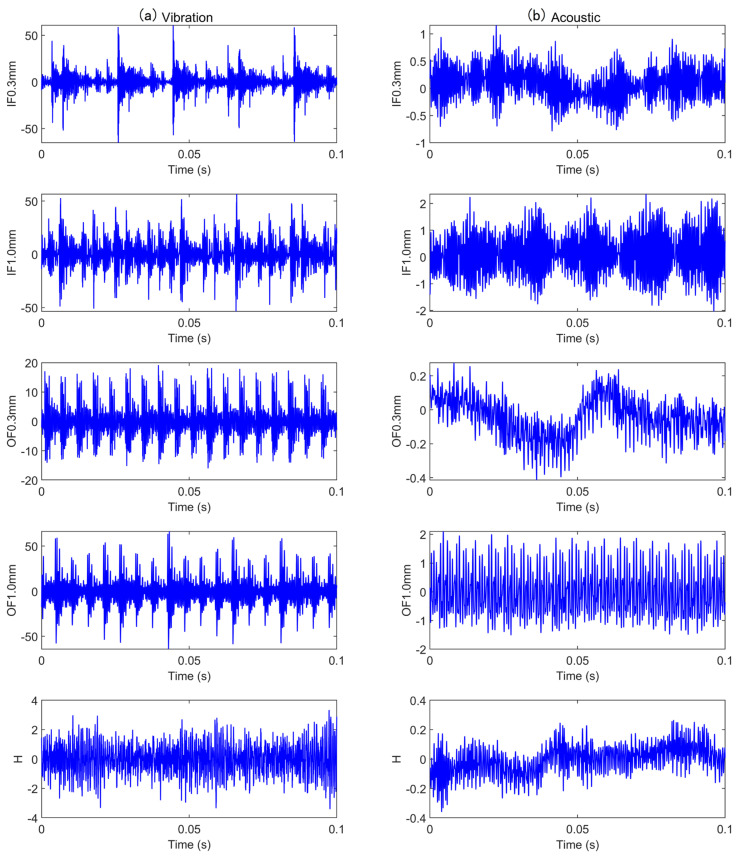
Original signals for various faults in case 2: (**a**) vibration signal; (**b**) acoustic signal.

The data we used was obtained from the bearing test bench at the Korea Advanced Institute of Science and Technology (KAIST) [45]. Collected from a rotating machinery test bench, which includes a three-phase asynchronous motor, a gearbox, a torque meter, a rotor, bearing housings A and B, and a hysteresis brake, as illustrated in Figure 16. The dataset uses standard NSK 6205 DDU bearings, operating at a rotational speed of 3010 rpm. The sampling frequency for the vibration signals is 25.6 kHz, while the sampling frequency for the acoustic signals is 51.2 kHz. The simulated loads are 0 Nm, 2 Nm, and 4 Nm, respectively. Accelerometers (PCB35234) are installed in the x and y directions on bearing housings A and B to collect vibration signals under different operating conditions. A microphone (PCB378B02) is installed near bearing housing A to collect acoustic data under zero-load conditions, since the air-cooled brake generates noise that may interfere with the microphone. Vibration and acoustic data were collected for five different states, categorized by crack damage diameter size: 0.3mm and 1.0mm. The faults simulated include inner race and outer race faults, resulting in three different bearing states (including the healthy state), thus a total of five bearing operating conditions. The raw vibration and acoustic signals for the five states are illustrated in Figure 17. Additionally, each operating condition incorporated 250 training samples and 50 testing samples. A detailed description of the sampling data for each condition is provided in Table 7.

#### 4.2.2. Effectiveness of the Proposed Method in Case 2

**Figure 18:**
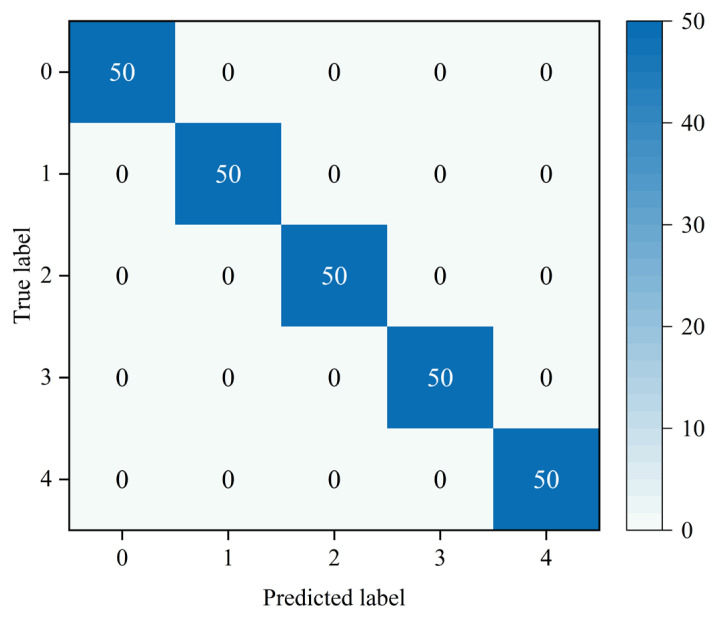
Confusion matrix of our proposed method in case 2.

**Figure 19:**
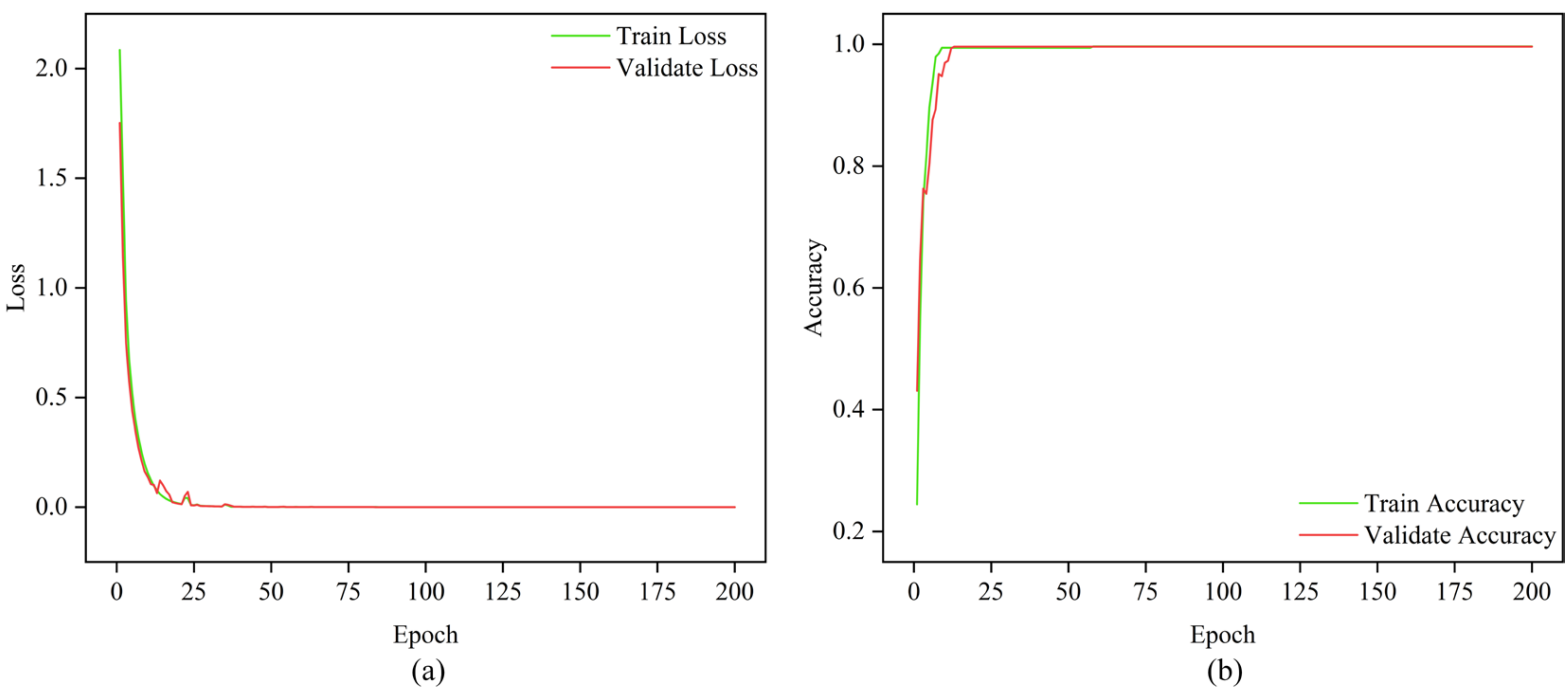
Iteration curve in case 2: (**a**) loss; (**b**) accuracy.

**Figure 20:**
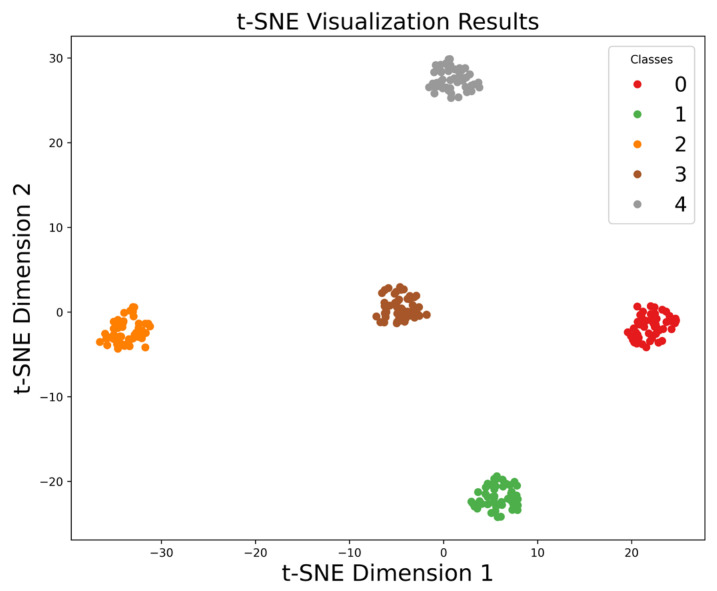
t-SNE visualization results of our proposed method in case 2.

In this section, the hyperparameter settings for MMSTCCAFN are consistent with those in case 1, and the fault diagnosis process used is also the same as in case 1. First, training samples are used to train the model, while test samples evaluate the fault diagnosis performance of MMSTCCAFN. Figure 18 shows the test sample confusion matrix. The predicted results match the actual labels, indicating that the predictions for each category are accurate. Figure 19 presents the Loss Curve and Accuracy Curve for 200 iterations, which stabilize after a few iterations. Figure 20 provides the t-SNE visualization, revealing complete class-wise sample clustering with well-defined boundaries, indicating the reliability of MMSTCCAFN in fault diagnosis for case 2.

**Figure 21:**
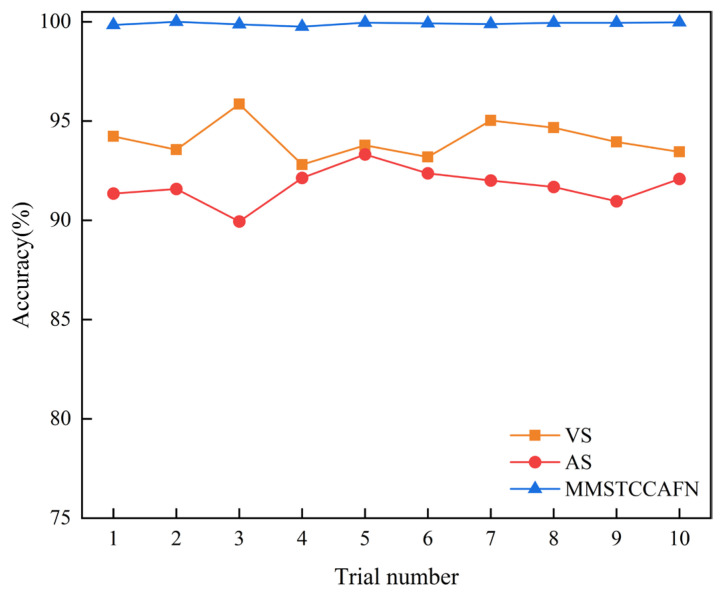
Comparison of inputs from different sensors in case 2.

Meanwhile, to highlight the benefits of multi-sensor fusion diagnosis, as mentioned earlier, MMSTCCAFN is also compared with the single sensors VS and AS. The statistical results from 10 trials for each sensor are shown in Table 8, with detailed outcomes presented in Figure 21. Clearly, using both vibration and acoustic sensors enables MMSTCCAFN to achieve an average accuracy of 99.91%, which is 5.86% and 8.17% higher than that of VS and AS, respectively. This further validates that MMSTCCAFN can effectively integrate critical fault features from multi-sensor signals, enhancing diagnosis accuracy and robustness.

#### 4.2.3. Ablation Experiments for Case 2

**Figure 22:**
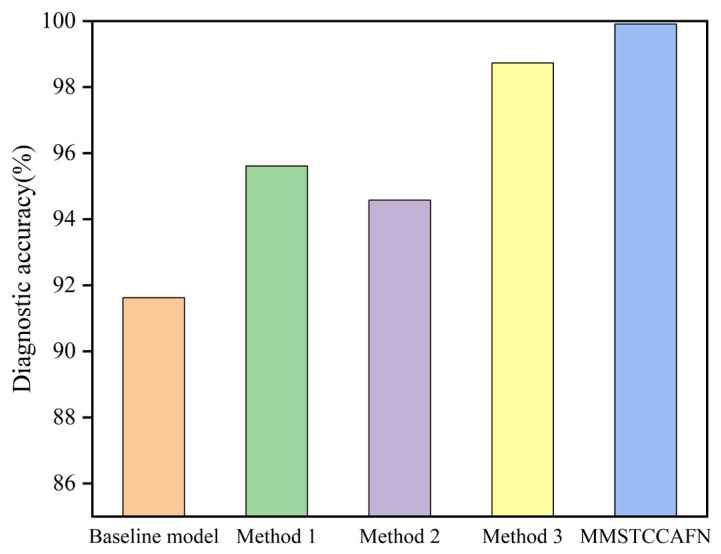
Ablation experiments in case 2: comparison of the diagnostic accuracy of different methods.

To comprehensively evaluate the proposed method’s influence on MMSTCCAFN performance and validate the architectural rationality of the fusion method, several ablation experiments were conducted on the dataset in case 2. Table 4 illustrates structural configurations of different ablation methods, while ablation experiment diagnostic accuracy results are depicted in Figure 22 and Table 9. The results indicate that the diagnostic performance of MMSTCCAFN exceeds that of Method 1, Method 2, and Method 3. Specifically, compared to Method 1, the use of MTCM improves recognition accuracy by 4.30% and reduces the standard deviation by 0.43, which can be attributed to MTCM’s robust and consistent feature extraction capabilities. Compared with Method 2, MFE enhances the ability to capture local and global contextual information and reduces redundant information, resulting in a 5.33% accuracy improvement and a 0.79 standard deviation reduction for MMSTCCAFN. Compared with Method 3, the use of DCA makes the fused features more complementary and improves the learning capability. The baseline model shows unstable performance compared to other methods and fails to achieve acceptable results. Through the mutual collaboration of the three modules, MMSTCCAFN demonstrates substantial performance enhancement in both accuracy and stability metrics.

#### 4.2.4. Comparison with Other Methods in Case 2

**Figure 23:**
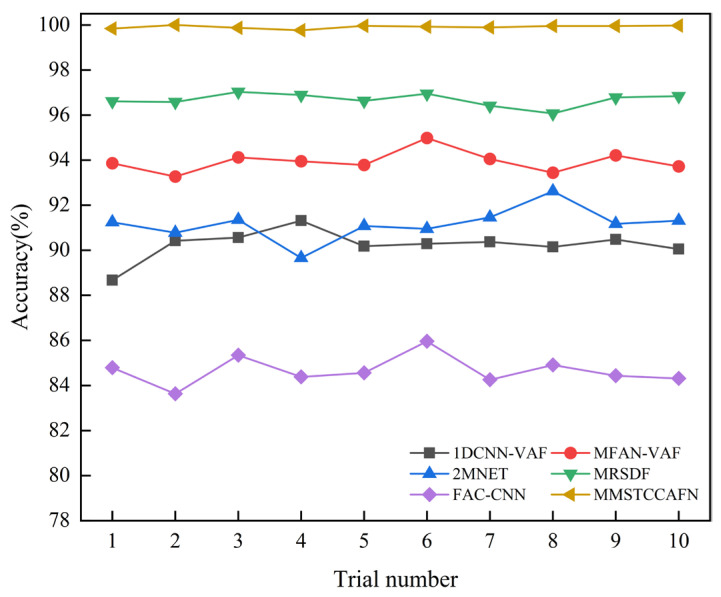
The comparison results of different architectures in case 2.

**Figure 24:**
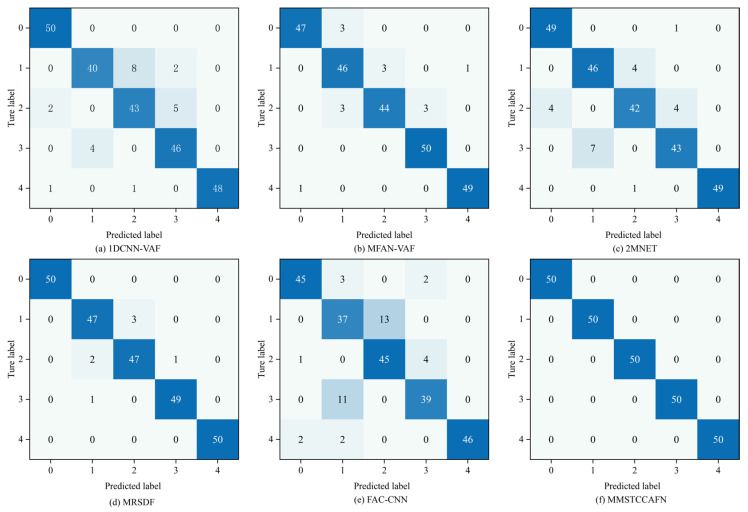
Confusion matrix of different methods in case 2: (**a**) 1DCNN-VAF; (**b**) MFAN-VAF; (**c**) 2MNET; (**d**) MRSDF; (**e**) FAC-CNN; (f) MMSTCCAFN.

To further validate the superiority of MMSTCCAFN, five fusion methods (1DCNN-VAF, MFAN-VAF, 2MNET, MRSDF, FAC-CNN) were again used for comparison. The parameters for MMSTCCAFN remained the same as in case 1. Similarly, to guarantee reliable diagnostic outcomes, the performance of each method was validated using 10 trials. The statistical outcomes of each method across the 10 trials are shown in Table 10, with detailed outcomes presented in Figure 23. The results indicate MMSTCCAFN achieves an optimal average accuracy of 99.91% and a minimal standard deviation of 0.0722, still outperforming the five comparison approaches. This further demonstrates the superiority and stability of MMSTCCAFN. Additionally, the diagnostic performance of the aforementioned methods for five different bearing conditions was analyzed using confusion matrices, as illustrated in Figure 24. The results demonstrate that significant overlaps exist in the classifications by 1DCNN-VAF, FAC-CNN, and 2MNET. In contrast, MMSTCCAFN showed no misclassifications across the five bearing conditions, further proving its superiority.

## 5. Conclusions

This paper proposes a multi-channel multi-scale spatiotemporal convolutional cross-attention fusion network (MMSTCCAFN) for bearing fault diagnosis. This network leverages the complementary characteristics and correlations between acoustic and vibration signals, performing adaptive fusion from multi-scale feature perspectives. As a result, it captures multi-level detailed information, enhancing diagnostic performance in high-noise environments while overcoming the potential limitations of relying on a single sensor. We validated the proposed method’s effectiveness using a comparison of diagnostic results from two bearing datasets with those from single sensors. Furthermore, compared to other methods, MMSTCCAFN achieves the highest average accuracy and the lowest standard deviation across both datasets, demonstrating its excellent performance and robustness. Additionally, the ablation experiments demonstrate the mutual cooperation of the three modules, highlighting the MMSTCCAFN’s commendable performance in terms of accuracy and stability.

Despite the high diagnostic accuracy of our proposed method, there are still certain limitations. Firstly, in practical industrial environments, additional data types such as speed, load, and temperature are also available. In this study, only vibration and acoustic data were fused. In the future, we plan to incorporate additional types of data to obtain more useful information. Secondly, despite the high diagnostic accuracy, the internal learning process of the method is complex, making it difficult to comprehend why the model arrived at a specific result. Future work will focus on enhancing the transparency and interpretability of the model by evaluating the final contributions of each input feature. Additionally, while the proposed method has potential advantages for real-time monitoring, practical implementation requires addressing issues such as sensor data synchronization and real-time data processing to ensure system stability and reliability. In our future research, we will prioritize solving problems related to computational complexity and system integration to enhance the practical application value of the method.

## Figures and Tables

**Table 1:** Details of training and test sets in case 1.

Bearing Health Condition	Number of Training Samples	Number of Test Samples	Labels
Healthy (H)	350	50	0
Developing fault (inner race) (DIF)	350	50	1
Faulty (inner race) (IF)	350	50	2
Developing fault (outer race) (DOF)	350	50	3
Faulty (outer race) (OF)	350	50	4
Developing fault (ball) (DBF)	350	50	5
Faulty (ball) (BF)	350	50	6
Developing fault (cage) (DCF)	350	50	7
Faulty (cage) (CF)	350	50	8

**Table 2:** The hyperparameters of the proposed method.

Hyperparameters	Parameter
Batch size	32
Epoch	200
Activation function	ReLU
Optimize	Adam
Learning rate	0.001

**Table 3:** Statistical results of inputs from different sensors in case 1.

Methods	Lowest Accuracy	Highest Accuracy	Average Accuracy	Standard Deviation (SD)
VS	92.46%	95.05%	93.70%	0.9316
AS	89.64%	92.36%	91.02%	0.9918
MMSTCCAFN	99.30%	99.64%	99.56%	0.0993

**Table 4:** Structure and parameters of MMSTCCAFN.

Method	ResNet	MTCM	MFE	DCA	Summation Fusion
Baseline model	√				√
Method 1	√		√	√	
Method 2	√	√		√	
Method 3	√	√	√		√
MMSTCCAFN	√	√	√	√	

**Table 5:** Diagnosis accuracy results of ablation experiments in case 1.

Method	Lowest Accuracy	Highest Accuracy	Average Accuracy	Standard Deviation (SD)
Baseline model	88.55%	92.37%	90.06%	1.3051
Method 1	94.08%	95.95%	95.10%	0.6234
Method 2	94.64%	96.12%	95.78%	0.4299
Method 3	97.80%	98.79%	98.16%	0.3392
MMSTCCAFN	99.30%	99.64%	99.56%	0.0993

**Table 6:** Statistical results of different architectures in case 1.

Methods	Lowest Accuracy	Highest Accuracy	Average Accuracy	Standard Deviation (SD)
1DCNN-VAF	91.20%	94.38%	93.54%	0.9060
MFAN-VAF	96.20%	97.06%	96.43%	0.2369
2MNET	90.27%	92.49%	91.05%	0.6273
MRSDF	94.25%	96.42%	95.29%	0.5557
FAC-CNN	80.33%	83.04%	82.37%	0.7984
MMSTCCAFN	99.30%	99.64%	99.56%	0.0993

**Table 7:** Details of training and test sets in case 2.

Bearing Health Condition	Size of Failure(mm)	Number Of Training Samples	Number of Test Samples	Labels
Healthy (H)	-	250	50	0
Inner race (IF)	0.3mm	250	50	1
	1.0mm	250	50	2
Outer race (OF)	0.3mm	250	50	3
	1.0mm	250	50	4

**Table 8:** Statistical results of inputs from different sensors in case 2.

Methods	Lowest Accuracy	Highest Accuracy	Average Accuracy	Standard Deviation (SD)
VS	92.81%	95.85%	94.05%	0.9176
AS	89.94%	93.32%	91.74%	0.8993
MMSTCCAFN	99.76%	100%	99.91%	0.0722

**Table 9:** Diagnosis accuracy results of ablation experiments in case 2.

Method	Lowest Accuracy	Highest Accuracy	Average Accuracy	Standard Deviation (SD)
Baseline method	90.33%	93.68%	91.62%	1.0277
Method 1	95.23%	96.72%	95.61%	0.5007
Method 2	93.29%	95.81%	94.58%	0.8577
Method 3	98.37%	99.16%	98.73%	0.2354
MMSTCCAFN	99.76%	100.00%	99.91%	0.0722

**Table 10:** Statistical results of different architectures in case 2.

Methods	Lowest Accuracy	Highest Accuracy	Average Accuracy	Standard Deviation (SD)
1DCNN-VAF	88.67%	91.31%	90.25%	0.6556
MFAN-VAF	93.27%	94.98%	93.94%	0.4688
2MNET	89.66%	92.62%	91.16%	0.7251
MRSDF	96.07%	97.03%	96.68%	0.2863
FAC-CNN	83.63%	85.96%	84.66%	0.6433
MMSTCCAFN	99.76%	100%	99.91%	0.0722

## Data Availability

The data are available from the corresponding author on reasonable request.
